# Enhancing ectasia screening using advanced AS-OCT: a case series of challenging refractive candidates

**DOI:** 10.3389/fopht.2024.1405443

**Published:** 2024-06-11

**Authors:** Niklas Mohr, Stefan Kassumeh, Nikolaus Luft, Martin Dirisamer, Siegfried G. Priglinger, Wolfgang J. Mayer

**Affiliations:** ^1^ Department of Ophthalmology, University Hospital, Ludwig-Maximilians-Universität, Munich, Germany; ^2^ Smile Eyes Clinic, Linz, Austria

**Keywords:** ectasia screening, AS-OCT, epithelial mapping, keratorefractive surgery, corneal tomography

## Abstract

**Purpose:**

Ectasia screening in candidates for laser refractive surgery is mandatory during preoperative evaluation. Despite the availability of modern imaging techniques, refractive surgeons often face borderline decisions when patients present with suspicious tomographic findings. This case series presents refractive candidates with suspicious tomographic findings and demonstrates how to interpret them using Scheimpflug imaging and additional anterior segment optical coherence tomography (AS-OCT).

**Setting:**

Department of Ophthalmology, University Hospital, LMU Munich.

**Case series:**

This case series examines six potential candidates for refractive surgery with a mean age of 29.2 ± 3.9 years, whose corneal assessments using Scheimpflug imaging raised suspicion for ectasia. Each candidate was additionally examined with AS-OCT and reevaluated. The mean manifest subjective spherical equivalent was -3.67 ± 1.8 diopters. The total corneal thickness measured 537 µm ± 30 µm at its thinnest point. None of the candidates had any reported underlying corneal or ophthalmic diseases, and slit lamp examinations revealed no abnormal morphological findings.

**Conclusions:**

Both Scheimpflug imaging and AS-OCT are appropriate tools for screening refractive candidates for ectasia. While topographic and elevation analyses yielded comparable results regarding corneal structure, the epithelial mapping provided by AS-OCT played a critical role in decision-making for cases with borderline tomographic findings. Establishing a global consensus on the use of epithelial mapping in ectasia screening is necessary.

## Introduction

Myopia is the leading refractive error, with a growing prevalence (1, 2). Consequently, the number of potential candidates for refractive surgery is increasing. While established techniques for refractive surgery have proven their safety and efficacy, preoperative patient evaluation and the selection of the most suitable procedure for each individual are fundamental to maintaining high standards, achieving satisfying results, and avoiding postoperative complications (3). Unfortunately, patients still experience iatrogenic ectasia after corneal refractive surgery, with an estimated incidence ranging from 0.01% to 0.6%, depending on the type of keratorefractive procedure (4, 5). Retrospective analyses revealed preoperative risk factors such as abnormal corneal topography, residual stroma bed thickness, age, and preoperative corneal thickness, as stated by Randleman in 2008 (6). Using the authors’ quantitative risk factor assessment, 91% of patients who developed post-keratorefractive surgery ectasia were retrospectively identified as “high-risk” candidates. As underlying pathogenesis, a combination of severe impacts on the corneal structure and alterde peroperative biomechanical properties are considered. Therefore, a weakness in the corneal structur must be exluded preoperatively. Thus, thorough preoperative corneal assessment is crucial for detecting early signs of ectatic disorders. Therefore, preoperative corneal assessment is crucial for detecting early signs of ectatic disorders (4).

Corneal imaging has tremendously evolved over the last decades (7). Modern devices now offer comprehensive full-thickness topography and tomography, including the corneal back surface, curvature maps, and elevation maps. Additionally, specialized tools like the Belin–Ambroísio enhanced ectasia display (BAD) simplify ectasia screening, allowing for the detection of ectatic alterations at early stages (8). A commonly used technique for refractive candidate evaluation is Scheimpflug imaging. Although the technology has improved over the years, there are ongoing developments in terms of software tools or automated data interpretation. These include deep learning algorithms that may surpass the performance of human clinicians in detecting and classifying keratoconus (9).

Anterior segment optical coherence tomography (AS-OCT) is emerging as a promising alternative to Scheimpflug imaging. Despite many similarities in corneal assessment techniques, such as topography and pachymetry, there are fundamental differences, notably in epithelial mapping, which is only available through non-contact AS-OCT or high-frequency ultrasound. In 2009, Reinstein has already reported on the early epithelial changes in ectatic corneas. Keratoconic eyes develop a typical apical epithelial thinning with surrounding “doughnut-shaped” epithelial thickening surrounding the keratoconic apex (10). This method appeared highly sensitive in detecting the early stages of keratoconic or ectatic eyes (11).

In a recent study, Asroui et al. (12) demonstrated the additive use of AS-OCT-based epithelial thickness mapping on the decision-making process for keratorefractive surgery candidates. Out of 100 patients, 10 patients initially excluded from the laser treatment were deemed suitable after additional epithelial map evaluation, while six patients previously considered suitable were excluded. This shows that in 16% of the cases, the decision was altered by incorporating epithelial mapping into the preoperative assessment for ectasia risk. Unfortunately, the precise methodology for assessing the epithelial maps was not published. Despite the available technology, refractive surgeons still face challenging cases.

For that purpose, we present a case series of six refractive candidates with debatable ectasia based on Scheimpflug imaging and demonstrate how these cases could be interpreted with additional AS-OCT and epithelial mapping, respectively.

## Patients and methods

This case series adhered to the principles of the Declaration of Helsinki. Institutional review board approval and consent from all participants were obtained for data analysis and publication.

The case series included six patients with an average age of 29 ± 4 years (range: 21–33 years) seeking refractive surgery consultation to achieve spectacle independence. In all cases, routinely performed Scheimpflug imaging obtained with the Pentacam HR device (Oculus Optikgeräte, Wetzlar, Germany) revealed suspicious ectasia findings. Subsequently, each patient underwent reevaluation using an AS-OCT system combined with Placido disc topography (MS-39; C.S.O., Florence, Italy) to map corneal epithelial thickness over a 9-mm zone.

As part of our routine procedure, patients were advised to discontinue wearing contact lenses for a minimum of 2 weeks before imaging. Both Pentacam and MS-39 examinations were performed three consecutive times each by an experienced examiner to ensure the validity of the results. The onboard quality assessment was carried out to verify the acceptable quality of each examination for it to be considered for evaluation. Immediately before the respective imaging, patients were instructed to blink to minimize potential distortion caused by the tear film. The assessment of the imaging data was performed by two experienced refractive surgeons (W.M. and N.L.).

Aside from the suspected ectasia, all candidates met the criteria for laser refractive surgery outlined by the German Committee of Refractive Surgery (“Kommission für refraktive Chirurgie”) (13). They had no history of eye diseases, dry eye syndrome, atopic dermatitis, eye injuries, or medication use. Slit lamp examinations showed regular anterior and posterior ocular segments in all patients. Preoperative manifest refraction spherical equivalent (MRSE) ranged from -1.0 D to -8.25 D (mean: -3.67 D ± 1.8 D), and pachymetry ranged from 501 µm to 584 µm (mean: 536.6 µm ± 29.5 µm).

## Case series

### Case 1

**Figure 1:**
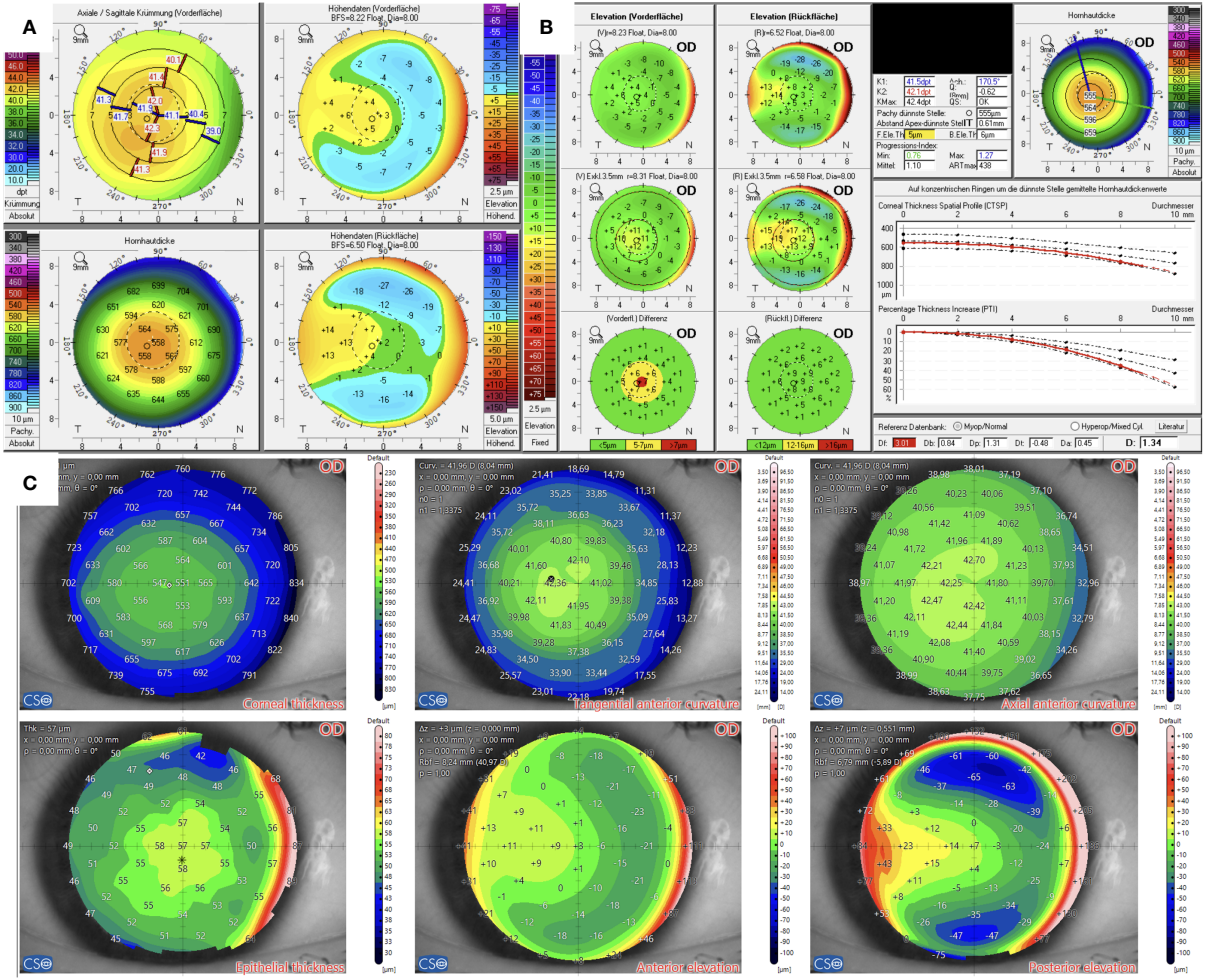
Tomographic findings of patient 1. **(A)** Refractive display by Pentacam HR, including axial anterior curvature (top left), corneal thickness (bottom left), anterior elevation (top right), and posterior elevation (bottom right). **(B)** Belin–Ambroísio display by Pentacam HR. **(C)** Corneal thickness (top left), epithelial thickness (bottom left), tangential (top center) and axial (top right) anterior curvature, anterior elevation (bottom center), and posterior elevation (bottom right) by MS-39.

The Pentacam examination of the candidate in Case 1 revealed protrusion of the corneal front surface in both eyes (**Figure 1**), as seen on the anterior surface elevation maps. There was also mild inferior topographic steepening and a mildly skewed radial axis. Manifest subjective refraction showed mild myopia with -2.75 D spherical equivalent and a best-corrected visual acuity (BCVA) of 20/15 in both eyes. The BAD displayed a suspicious anterior surface elevation profile, where the “final D” was regular. Contrary to the Pentacam, MS-39 indicated no abnormal findings with a regularly distributed epithelial thickness, with the thickest point located centrally. Elevation maps were classified as without pathological findings. The onboard screening tool for keratoconus also indicated normal corneas for both eyes.

### Case 2

**Figure 2:**
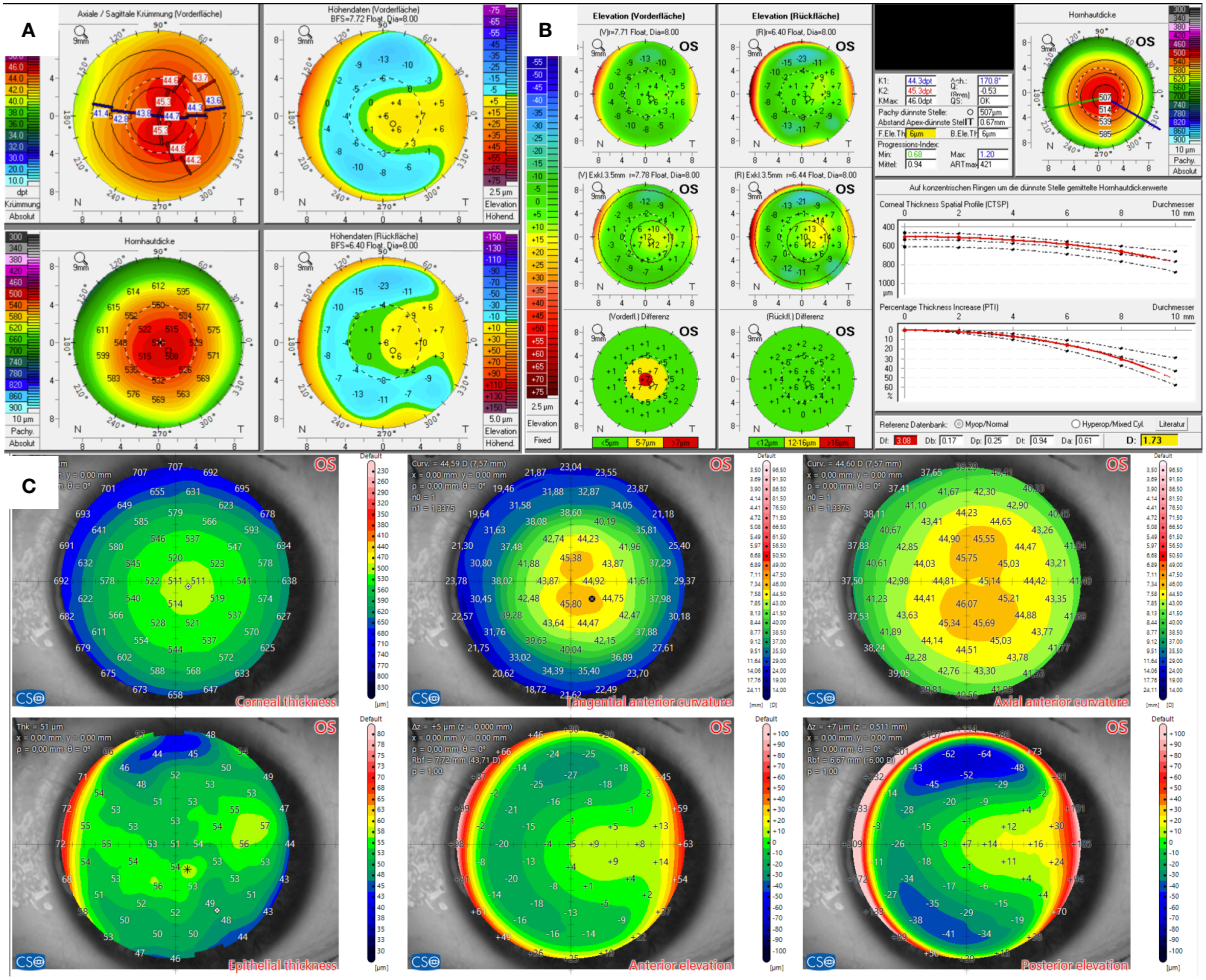
Tomographic findings of patient 2. **(A)** Refractive display by Pentacam HR, including axial anterior curvature (top left), corneal thickness (bottom left), anterior elevation (top right), and posterior elevation (bottom right). **(B)** Belin–Ambroísio display by Pentacam HR. **(C)** Corneal thickness (top left), epithelial thickness (bottom left), tangential (top center) and axial (top right) anterior curvature, anterior elevation (bottom center), and posterior elevation (bottom right) by MS-39.

The candidate in Case 2 (**Figure 2**) had anisometropia with moderate myopia in the right eye of -4.75 D spherical equivalent and high myopia in the left eye of -8.25 D spherical equivalent, with suspicion for mild amblyopia. Visual acuity was 20/25 in the right eye and 20/30 in the left eye. The subjective manifest refraction revealed mild astigmatism of -1.5 D and -2.0 D as well. The thinnest point measured by Pentacam in both eyes was 507 µm, localized almost centrally. Elevation maps implied a central protrusion with a partial doughnut pattern, especially on the front surface. A topographic map showed a skewed radial axis. The yellow-highlighted final D value of 1.73 of the BAD indicated suspicion for keratoconus.

In comparison, AS-OCT confirmed thin pachymetry but only showed non-specific alterations in the elevation maps without significance, as shown for the left eye in **Figure 2B**). The physiological epithelial thickening appears to be most pronounced slightly inferior to the corneal vertex. However, the epithelium thickens at the corresponding area of corneal protrusion in question. The onboard screening tool for keratoconus also indicated normal corneas.

### Case 3

The candidate in Case 3 presented with mild myopia. Pentacam’s BAD indicated abnormal front surfaces for both eyes. Additionally, the right eye showed a doughnut pattern in the elevation map and a conspicuous corneal thickness spatial profile (CTSP). The total corneal thickness was 515 µm in the right eye and 512 µm in the left eye at the thinnest point. Visual acuity was 20/20 in both eyes, with a manifest subjective refraction of -1.75 D and -1.0 D spherical equivalent.

**Figure 3:**
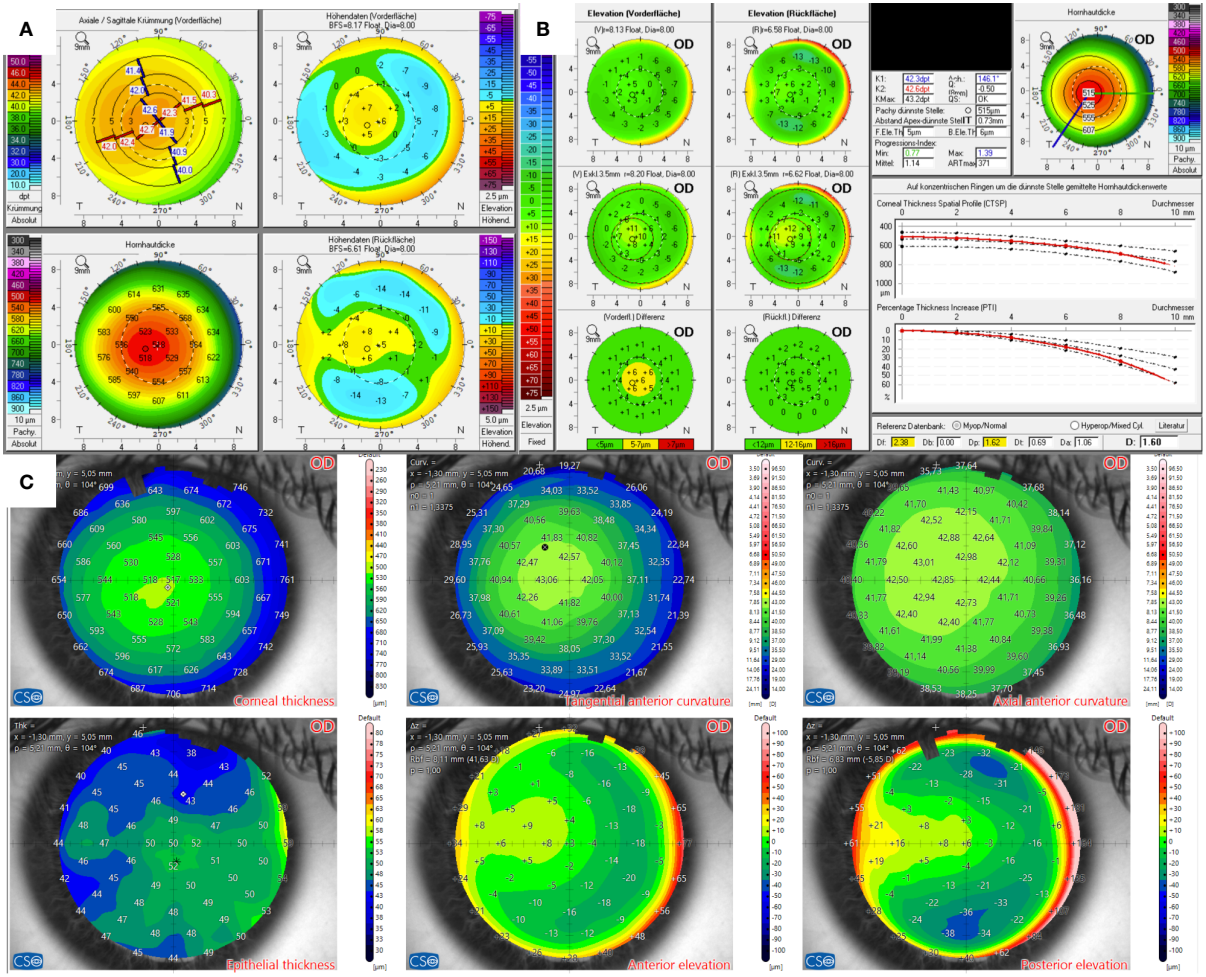
Tomographic findings of patient 3. **(A)** Refractive display by Pentacam HR, including axial anterior curvature (top left), corneal thickness (bottom left), anterior elevation (top right), and posterior elevation (bottom right). **(B)** Belin–Ambroísio display by Pentacam HR. **(C)** Corneal thickness (top left), epithelial thickness (bottom left), tangential (top center) and axial (top right) anterior curvature, anterior elevation (bottom center), and posterior elevation (bottom right) by MS-39.

In contrast, AS-OCT’s elevation maps were regular, as were the epithelial maps, as shown in **Figure 3**. The epithelial map of both eyes showed a thinning in almost the entire superior hemisphere, with the center being the thickest point. The onboard screening tool for keratoconus also indicated normal corneas.

### Case 4

**Figure 4:**
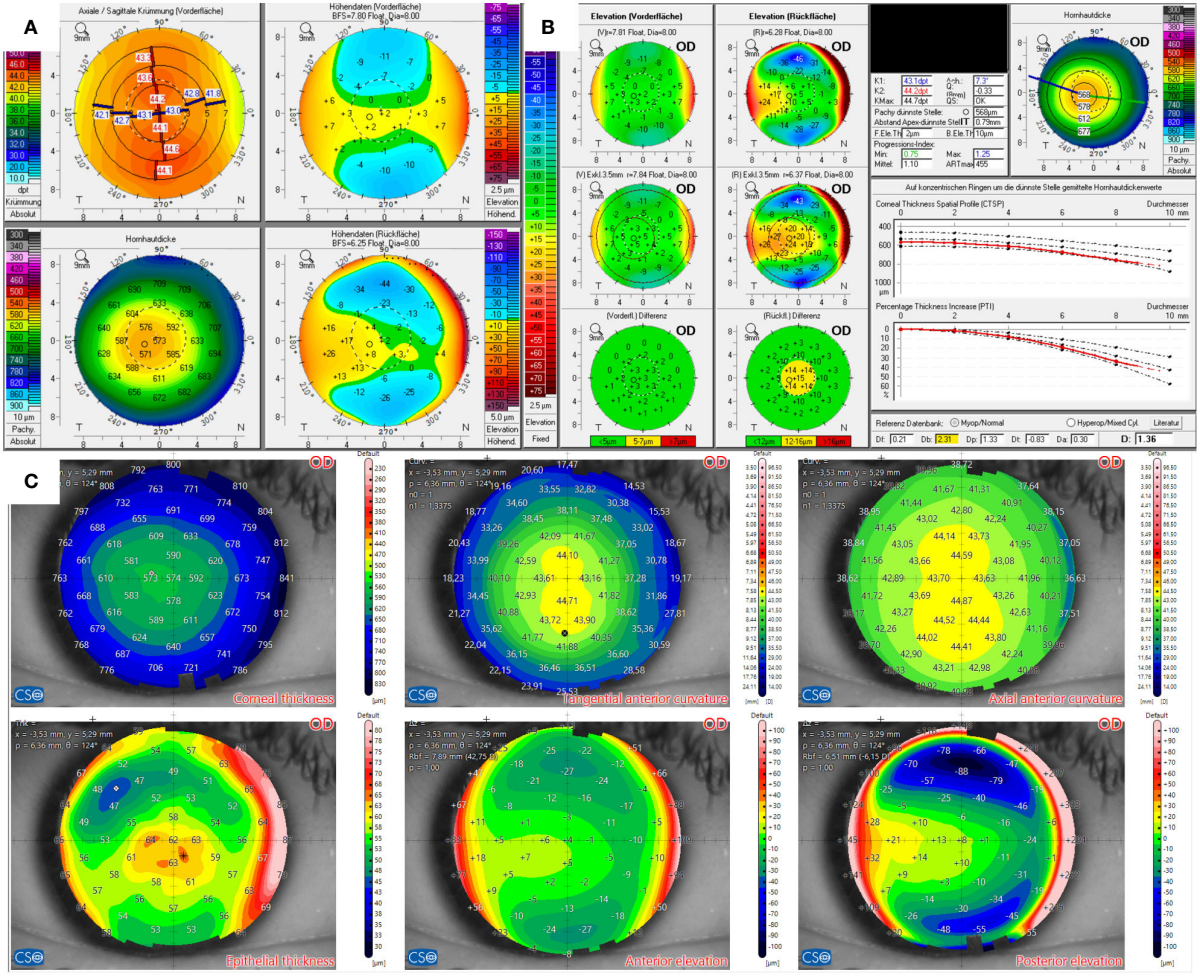
Tomographic findings of patient 4. **(A)** Refractive display by Pentacam HR, including axial anterior curvature (top left), corneal thickness (bottom left), anterior elevation (top right), and posterior elevation (bottom right). **(B)** Belin–Ambroísio display by Pentacam HR. **(C)** Corneal thickness (top left), epithelial thickness (bottom left), tangential (top center) and axial (top right) anterior curvature, anterior elevation (bottom center), and posterior elevation (bottom right) by MS-39.

The Scheimpflug examination of the right eye of the candidate in Case 4, as displayed in **Figure 4**, revealed minor topographic inferior steepening and a conspicuous elevation map of the posterior surface in the BAD. The thinnest point of the total cornea measured 568 µm. The candidate exhibited mild to moderate myopia with a low astigmatic component. The manifest subjective refraction was -3.0 D sph. -0.5 D cyl. 172° in the right eye and -2.5 D sph. -0.75 D cyl. 178° in the left eye, with a BCVA of 20/15.

On the contrary, AS-OCT showed epithelial thickening paracentrally inferior, which was consistent with the mild inferior steepening of the anterior surface. Hence, the epithelial thickening was considered the cause of the topographic alterations. Automated keratoconus screening classified the cornea as normal.

### Case 5

**Figure 5:**
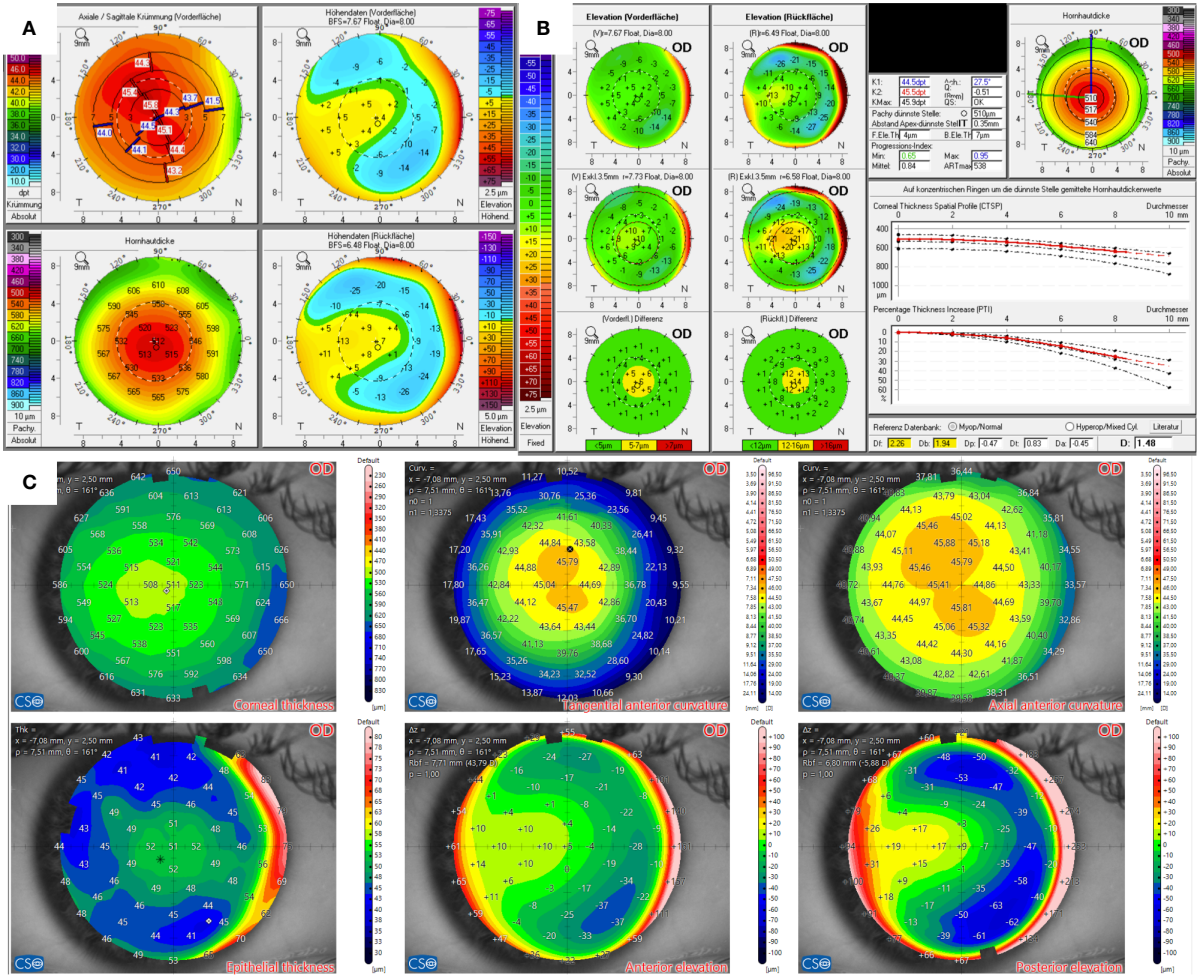
Tomographic findings of patient 5. **(A)** Refractive display by Pentacam HR, including axial anterior curvature (top left), corneal thickness (bottom left), anterior elevation (top right), and posterior elevation (bottom right). **(B)** Belin–Ambroísio display by Pentacam HR. **(C)** Corneal thickness (top left), epithelial thickness (bottom left), tangential (top center) and axial (top right) anterior curvature, anterior elevation (bottom center), and posterior elevation (bottom right) by MS-39.

In Case 5 (**Figure 5**), the candidate had moderate myopia of -4.5 D in the right eye and -5.25 D in the left eye. His total cornea’s thinnest point, measured by Pentacam, was 510 µm and 501 µm in the right eye and left eye, respectively. Elevation maps indicated central protrusion of both the front and back corneal surfaces. Hence, D values for the front and back (Df and Db) surfaces reached a range of suspicious values, as highlighted in yellow. However, the final D values remained within a normal range. By comparison, AS-OCT confirmed the thin pachymetric values and showed slight protrusion in the elevation maps of both eyes, as well. This slight protrusion was located superiorly temporally. The epithelial mapping revealed a normal distribution, with the thickest area in the center and thinning in the periphery, although the epithelium was significantly thinned overall. A screening tool for keratoconus indicated normal corneas. In summary, Pentacam’s findings suggested a suspicious cornea, while MS-39 indicated almost normal corneas except for the peripherally emphasized thinned epithelium. The thinned epithelium pattern could also be caused by lens warpage, potentially masking underlying ectatic alterations. Consequently, the patient was deemed unsuitable for laser refractive surgery, as ectasia could not be safely excluded. Phakic intraocular lenses may be considered as an alternative option. Therefore, progressive corneal ectasia should be ruled out by further follow-up examinations.

### Case 6

**Figure 6:**
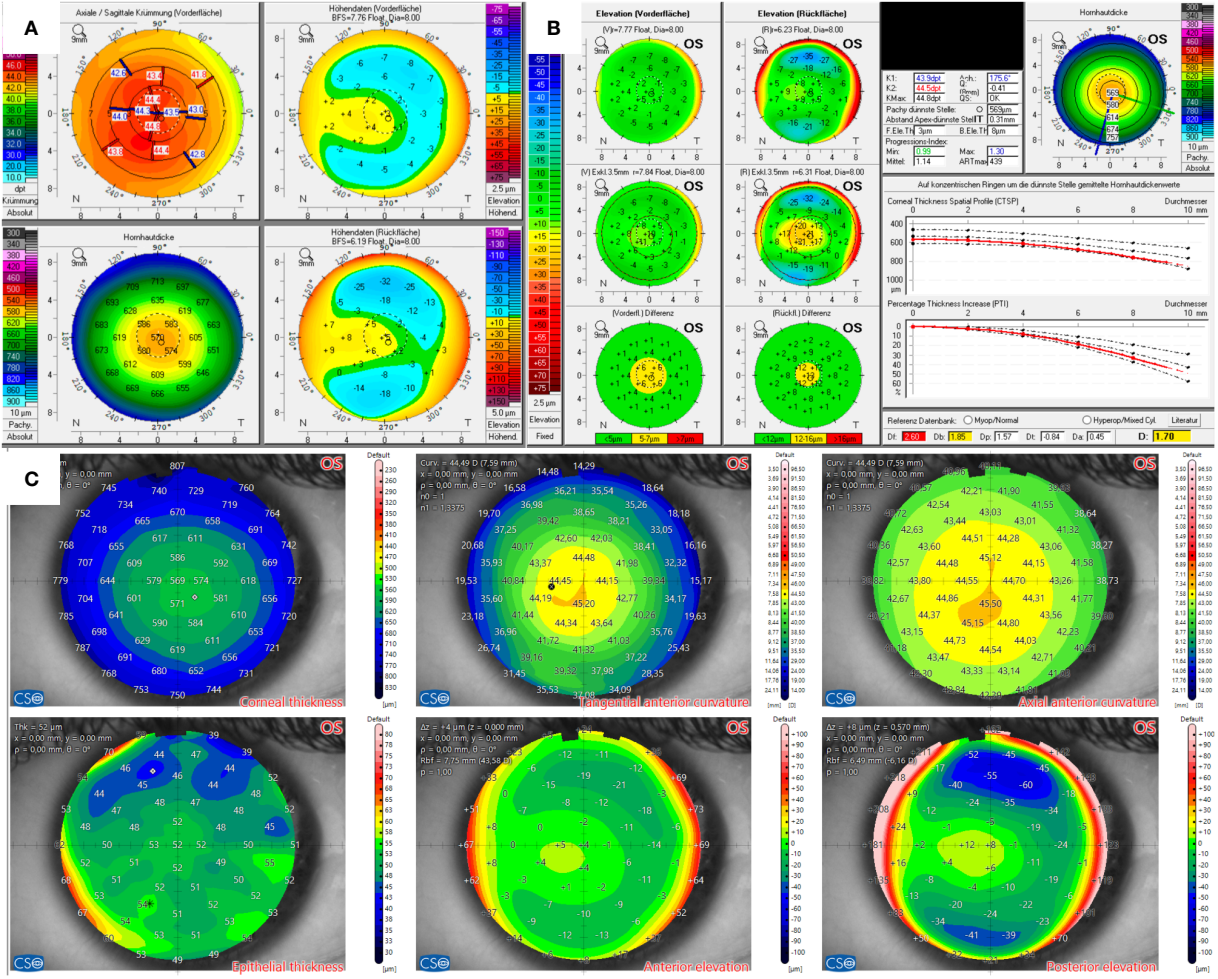
Tomographic findings of patient 6. **(A)** Refractive display by Pentacam HR including axial anterior curvature (top left), corneal thickness (bottom left), anterior elevation (top right), and posterior elevation (bottom right). **(B)** Belin–Ambroísio display by Pentacam HR. **(C)** Corneal thickness (top left), epithelial thickness (bottom left), tangential (top center) and axial (top right) anterior curvature, anterior elevation (bottom center), and posterior elevation (bottom right) by MS-39.

**Figure 6** displays the Pentacam and MS-39 findings of the left eye of candidate 6, who had mild to moderate myopia with -3.5 D on the right and -3.0 D on the left eye. The Pentacam’s BAD indicated suspicious posterior surfaces in both eyes. Additionally, the corneal thickness spatial profile (CTSP) was abnormal in the right eye, while the left eye also showed abnormal front surface elevation. The thinnest corneal thickness measured 584 µm in the right eye and 569 µm in the left eye.

MS-39 showed normal topographic maps with mild protrusion in the elevation map of the corneal back surface in both eyes and localized inferonasal paracentral. However, the on-board screening tool for keratoconus indicated normal corneas in both eyes. The epithelium appeared marginally irregular, with localized thinning over the area of protrusion. In summary, the MS-39 corroborated the suspicion of ectasia, and the patient was declined as a laser refractive candidate.

## Discussion

Scheimpflug-based systems, such as the Pentacam HR, are commonly used to evaluate the corneas of potential candidates before refractive surgery. However, the presented cases have shown that there can be controversial results between the two devices for borderline findings. The final decision on whether a candidate is eligible for keratorefractive surgery depends on the interpretation and weighing of the various findings. In particular, for epithelial mapping, there is no standardized classification system or definition for normal or suspicious epithelium that a surgeon can use as reference.

In general, the corneal epithelium compensates for structural alterations of the underlying stroma. This epithelial remodeling leads to typical findings in ectatic corneas (14). Over the conical area with stromal thinning and protrusion, the epithelium thins to maintain a physiological curvature. Subsequently, the surrounding epithelium thickens. This epithelial thinning with “doughnut-shaped” thickening was first described by Reinstein et al. using the VHF-US (Artemis) for keratoconic eyes (15). In further studies, it was proven that these changes also appeared in very early stages and were therefore beneficial in keratoconus screening as an early indicator (16, 17). However, it is not described at what amount or expansion of thinning the epithelial pattern can be defined as keratoconic.

The localized epithelial thinning can occur with or without a surrounding circular thickening, as reported recently by Levy et al. (18). It was also described that keratoconic eyes exhibit central epithelial thickening or peripheral annular thinning (18). Due to the epithelium responding to several corneal or environmental alterations in order to maintain the corneal surface, it is vulnerable to external factors that could mask ectatic profiles. For example, dry eyes can lead to superior thinning and inferior thickening (18). Lens warpage is caused by the direct mechanical effect of the contact lens, leading to central thickening and surrounding thinning (19), creating an epithelial profile that is opposite to the keratoconic pattern. Additionally, central thickening can also arise in cases of epithelial basement membrane dystrophy (20). A unique pattern of keratoconus, recently named by Yang et al., is the coincident thinning pattern on corneal and epithelial thickness maps and the concentric thinning pattern on the epithelial thickness map in AS-OCT (21).

However, there are still no accurate definitions or thresholds for normal or abnormal epithelium., especially when it comes to borderline findings. In a study by Asroui et al. (12), a pattern of central or inferior thinning of epithelial thickness was defined as a pattern to increase the examiners’ concern about patients’ risk for ectasia. It can be assumed that the decisions were highly dependent on the individual examiners’ experience since there was no accurate definition of conspicuous epithelium. In summary, despite offering a remarkable benefit in diagnostics, epithelial mapping is still a supporting tool that should be interpreted in conjunction with other diagnostic findings.

In Cases 1–3, Pentacam findings only revealed front surface alterations with regular-shaped posterior surfaces. According to the global consensus on keratoconus published in 2015, posterior corneal elevation abnormalities must be present to diagnose early or subclinical keratoconus (22). Prior to refractive surgery, however, the goal is not only to diagnose and rule out an ectatic disorder but also to analyze the risk of developing ectasia after the surgery. Therefore, cases like 1–3 that do not show such posterior surface alterations have to be categorized as suspicious. Inferior steepening in Case 1 and the minor skewed axis raised suspicion but these could be associated with the angle kappa of the patient. Corneal thickness was above 550 µm at the thinnest point, and predicted stromal ablation was low. Additionally, the epithelial thickness map showed a regular profile with thickening corresponding to the area of inferior steepening. Therefore, the candidate was classified as eligible for Small-Incision-Lenticule-Extraction (SMILE) surgery.

The tomographic findings of the candidate in Case 2 also revealed a significant angle kappa, which resulted in a skewed radial axis in the topographic maps. The epithelial map showed areas of focal epithelial thickening in the inferior part of the cornea. As described earlier, dry eye can cause such changes in the epithelial profile. However, the epithelial map could also be interpreted as showing central epithelial thinning without a characteristic doughnut-shaped profile. Additionally, the potential thinning was only 2 microns. It is important to note that current AS-OCTs have an axial resolution of 3 µm to 4 µm (23), which is more than 5% of the total epithelial thickness, averaging about 55 µm at its thickest point, according to the literature. Interestingly, the epithelial map of MS-39 can be color-coded up to a 2-µm scale. Corneal thickness measured 507 µm at the thinnest point with a regular thickness profile (CTSP). Repeated imaging confirmed regular astigmatism, and the candidate was eventually approved for a SMILE procedure.

The corneal evaluation of the 21-year-old candidate in Case 3 revealed doughnut-shaped elevation maps and an anterior surface and thickness profile on the BAD. In contrast, the epithelial map showed a regular profile with superior thinning that may be associated with dry eye. The candidate was seeking spectacle independence as he required acceptable, uncorrected distance visual acuity for his profession. The subjective manifest refraction had been stable for the last 3 years. We decided to repeat the examinations 3 months later to rule out any progression. Eventually, the candidate underwent the LASIK procedure. The regular final D and strong symmetry of the fellow eye also played a role in the decision to perform keratorefractive surgery.

The only possible risk factor for post-laser ectasia in Case 4 was the protrusion of the posterior surface revealed by the elevation map and the BAD. In contrast, the final D was within regular margins, and the front surface was regular. The total corneal thickness profile was normal, with the thinnest point measuring 568 µm in both eyes. The expected stromal ablation, which is relatively low due to the mild myopia of the patient, must also be considered, as it leads to lower detriment to corneal stability. Additionally, the epithelial thickness map showed central thickening. It is noteworthy that Levy et al. (18) reported that 56% of patients with keratoconus exhibit a doughnut-specific pattern in the epithelial thickness map, and 47% of keratoconic patients have central epithelial thinning as the only abnormal epithelial feature. Unfortunately, it is not specified if and how these groups overlap. Conversely, only a few other conditions besides keratoconus elicited an epithelial doughnut pattern. They concluded with high specificity but moderate sensitivity. Therefore, the absence of typical epithelial changes reduces the likelihood of keratoconus. However, based on its low sensitivity, keratoconus may not be completely ruled out solely because of a normal epithelium. The transferability of these findings to ectasia risk analysis before refractive surgery has not yet been clarified. Nevertheless, the patient in Case 4 was ultimately approved for SMILE surgery because, in our view, the otherwise regular findings and the epithelial map outweighed the slight posterior surface protrusion.

In Case 5, the patient presented with a thin cornea, irregular anterior topography, and suspicious BAD readings on both the front and back surfaces. The epithelial map revealed peripheral annular thinning, which is atypical for keratoconus but not a normal epithelial profile. As described before, it may be caused by wearing contact lenses. However, it could mask an ectatic epithelial profile. Therefore, the patient should not be considered for laser refractive surgery, as ectasia cannot be safely ruled out. In cases where there is uncertainty about ectasia, combined treatments such as photorefractive surgery plus corneal crosslinking can be considered. Long-term results showed convincing outcomes in absence of adverse advents. Generally, a topography-guided profile is used for each patient individually, but standardized procedures are still lacking (24).

Although the pachymetry of the total cornea of the candidate in Case 6 was within normal ranges, the BAD indicated suspicious front and back surfaces. Corneal protrusion could also be seen in the elevation maps of the MS-39, localized to the inferotemporal paracentral area. In this area, the epithelial map revealed a small area of focal thinning, which corroborated the suspicion for ectasia. Even if the thinning is only a few micrometers, as discussed earlier, the candidate should be rejected for keratorefractive surgery as a precaution in the summary of the findings.

Limitations may be found in the present case series. Some patients were deemed eligible for laser refractive surgery based on diagnostic findings, but there was no long-term follow-up to confirm whether post-laser ectasia could occur. Typically, ruling out ectasia requires several years of follow-up. Reports in the literature have shown cases of post-LASIK ectasia even 10 years after treatment (25). The incidence of post-laser ectasia is rare and estimated between 0.01% and 0.6% (4, 5). Hence, for proper evaluation of the development of ectasia due to laser-refractive surgery, a large number of patients are needed. The current state of knowledge is based on retrospectively investigated cases of ectasia.

In conclusion, one of the main goals before laser refractive surgery is to rule out ectasia. Literature provides a wealth of data on patterns and findings for early recognition of ectatic diseases. However, there is currently no clinically established classification system for ectasia and there are no clear cut-off values for ectasia. In the end, it is an individual decision to be made by the surgeon for each patient. Therefore, the more information provided about the cornea, the more secure the decision will be. The presented cases show how epithelial mapping with AS-OCT can influence the surgeon’s decision in preoperative corneal assessments. Early corneal alterations in ectatic disorders, such as epithelial thinning over the cone, can be detected and topographic changes of the anterior surface caused by lens warpage or subepithelial corneal dystrophy can be revealed. Thus, a combination of different devices and imaging modalities should be considered in challenging cases. However, there are still no guidelines on how to interpret epithelial maps in detail and how they should be used in ectasia screening. Among other things, questions that need to be answered are the following: What is a normal and abnormal epithelium? Can “normal” epithelium outweigh suspicious tomography and thus rule out ectasia? Should epithelial mapping be used in refractive candidates regardless? What is the prognostic value of epithelial mapping in relation to ectasia? Eventually, future studies and a global consensus are needed, as the epithelial thickness map can significantly influence the surgeon’s decision.

## Data availability statement

The data that support the findings of this study are available from the corresponding author, NM, upon reasonable request. Requests to access these datasets should be directed to niklas.mohr@med.uni-muenchen.de.

## Ethics statement

The studies involving humans were approved by Ethics committee of the Ludwig-Maximilians-University. The studies were conducted in accordance with the local legislation and institutional requirements. Written informed consent for participation was not required from the participants or the participants’ legal guardians/next of kin in accordance with the national legislation and institutional requirements. Written informed consent was obtained from the individual(s) for the publication of any potentially identifiable images or data included in this article.

## Author contributions

NM: Conceptualization, Data curation, Formal analysis, Investigation, Methodology, Project administration, Resources, Software, Supervision, Validation, Visualization, Writing – original draft, Writing – review & editing. SK: Data curation, Investigation, Methodology, Validation, Writing – original draft. NL: Conceptualization, Supervision, Validation, Writing – review & editing. MD: Investigation, Project administration, Supervision, Validation, Writing – review & editing. SP: Conceptualization, Project administration, Resources, Supervision, Validation, Writing – review & editing. WM: Conceptualization, Data curation, Investigation, Methodology, Project administration, Resources, Supervision, Validation, Writing – original draft.
